# Carbon Nanotube Array Based Binary Gabor Zone Plate Lenses

**DOI:** 10.1038/s41598-017-15472-9

**Published:** 2017-11-10

**Authors:** Sunan Deng, Tahseen Jwad, Chi Li, David Benton, Ali K. Yetisen, Kyle Jiang, Qing Dai, Haider Butt

**Affiliations:** 1 Nanotechnology Laboratory, Department of Mechanical Engineering, University of Birmingham, Birmingham, B15 2TT UK; 2 Laboratory of Applied Photonics Devices, School of Engineering, École Polytechnique Fédérale de Lausanne, CH-1015 Lausanne, Switzerland; 3 National Center for Nanoscience and Technology, Beijing, 100190 China; 4 Aston Institute of Photonic Technologies, Aston University, Birmingham, B4 7ET UK; 5 Harvard-MIT Division of Health Sciences and Technology, Harvard University and Massachusetts Institute of Technology, Cambridge, MA 02139 USA

## Abstract

Diffractive zone plates have a wide range of applications from focusing x-ray to extreme UV radiation. The Gabor zone plate, which suppresses the higher-order foci to a pair of conjugate foci, is an attractive alternative to the conventional Fresnel zone plate. In this work, we developed a novel type of Beynon Gabor zone plate based on perfectly absorbing carbon nanotube forest. Lensing performances of 0, 8 and 20 sector Gabor zone plates were experimentally analyzed. Numerical investigations of Beynon Gabor zone plate configurations were in agreement with the experimental results. A high-contrast focal spot having 487 times higher intensity than the average background was obtained.

## Introduction

Diffractive zone plates have a myriad of applications in the short wavelength regime where ordinary glass is opaque^1–3^. They enable focusing x-ray and extreme UV radiation, such as x-ray microscopy using synchronous sources^4^, x-ray astronomy^5^, and UV spectroscopy^6,7^. The Fresnel zone plate (FZP), consisting of a series of transparent and opaque zones, is the most commonly used zone plate owing to its simple form for ease of fabrication. However, in comparison to refractive lenses, FZP suffers from degraded efficiency and high background noise as it has multiple focal points with focal lengths ${f}_{p}=\pm {r}_{1}^{2}/p\lambda$, $p=1,3,5\ldots$ ^5^, although the first order foci can in principle produce improved resolution.

The Gabor zone plate (GZP), which suppresses the higher-order foci to a pair of conjugate foci at $f=\pm {r}_{1}^{2}/\lambda$ ^5^, is an attractive alternative to the conventional FZP lens. However, the traditional GZP lens has been difficult to fabricate with the required sinusoidal transmission in each zone. In the 1990s, a binary GZP lens was proposed by inducing an azimuthal and radial variation of the transmittance^6^. Since the binary GZP has no higher order, it has high potential for optics applications such as Broglie matter wave optics and hard X-rays due to low-cost and facile production. Nevertheless, its fabrication is challenging due to the complex azimuthal transmission function^8–10^. Although binary GZP can be fabricated in a Kipp-inspired sieve configuration, it yields inefficient light focusing intensity^9^. Hence, the development of nanofabrication approaches to accurately produce binary GZPs is highly desirable for the development of efficient lenses.

A binary Gabor zone plate consists of transparent and opaque zones with respect to the incident irradiation which allows it to act as a lens. As for reflective binary zone plate, the reflections from the opaque zones will decrease the focusing efficiency^11–13^. Due to the high absorption of CNT array in both UV^14,15^ and visible regimes^16^ (reflectance can be up to 0.045% in visible regime), it will be a perfect material for the opaque areas in a Gabor zone plate. In the present work, we show the development of a binary GZP lens with CNT array. Three types of GZP lens were developed and their performance were optically characterized and compared to the literature values. With 650 nm incident light, the lenses can focus light with a spot intensity 487 times higher than the average background.

## Results and Discussions

**Figure 1:**
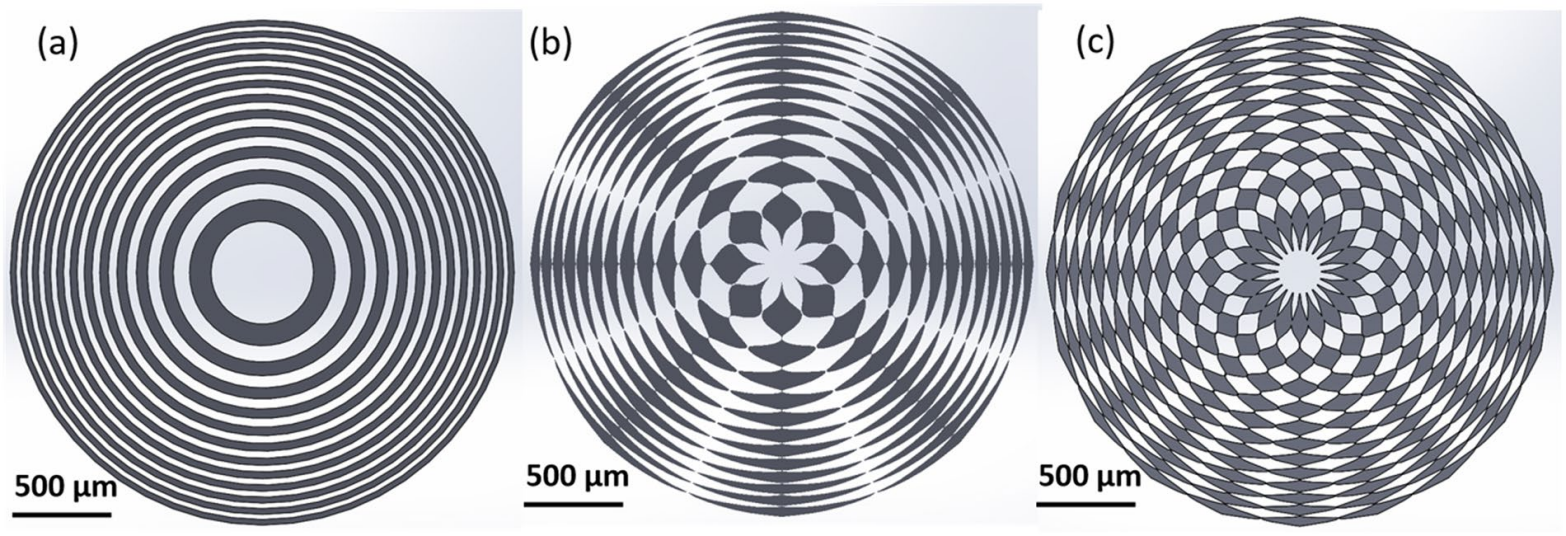
Schematic images of three types of GZP lens with (**a**) 0, (**b**) 8, and (**c**) 20 sectors. The radii of the central zone for the lenses were 250 µm, while the diameters of the lenses were 2.5 mm.

In the present work, three GZP lenses W = 0, 8, 20 separately were studied (Fig. 1). These lenses had the same size, with 250 µm for the first zone radius and 2.5 mm for the lens diameter.

**Figure 2:**
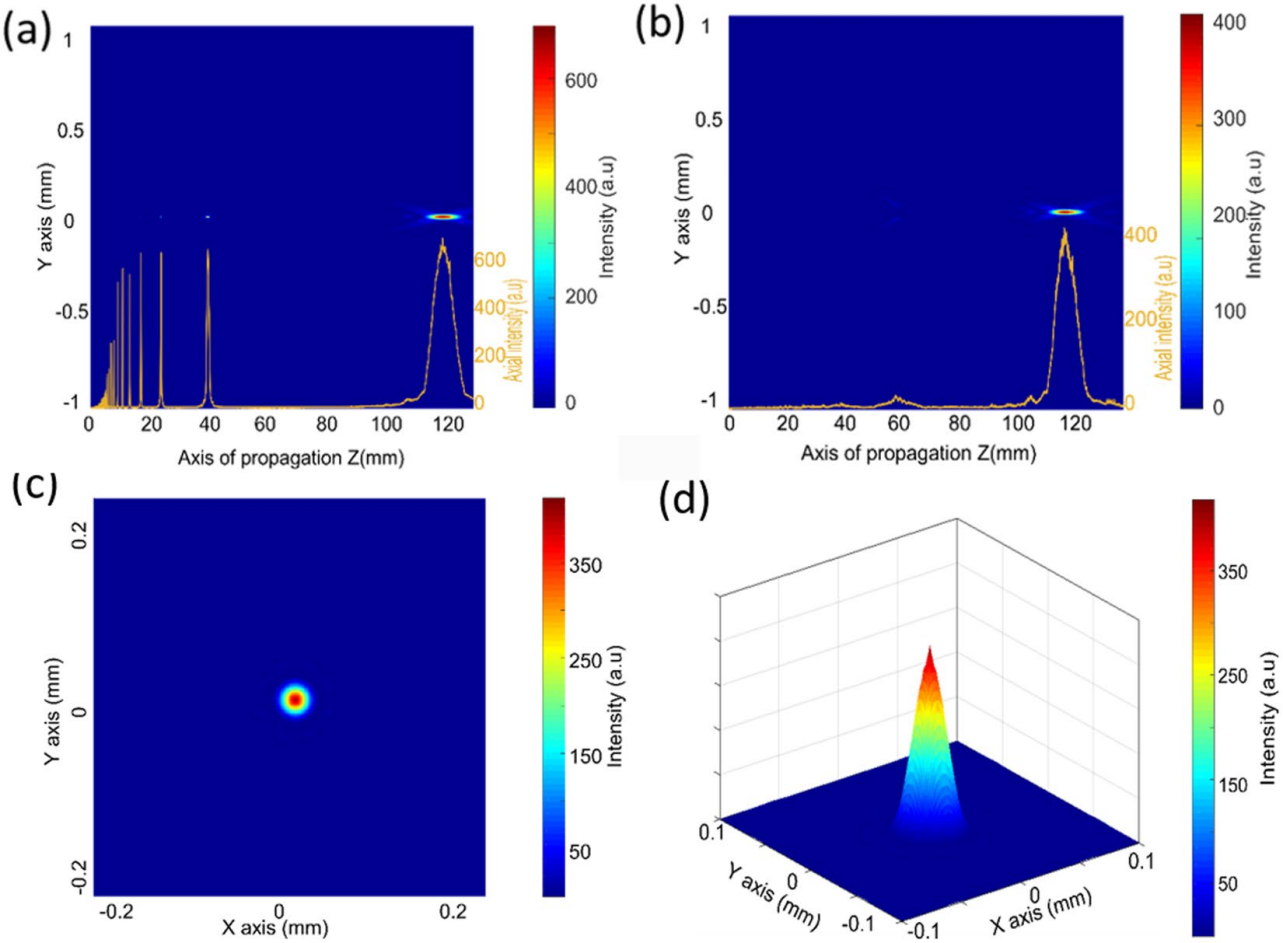
Intensity distribution and axial intensity along the axis of propagation of (**a**) 0 and (**b**) 8 sector GZP lens with 532 nm incident light, (**c)** focal plane of 8 sector GZP lens, and (**d**) 3D image of (**c**).

Figure 2a,b shows the simulation results about the intensity distribution and axial intensity along the axis of propagation of 0 and 8 sector GZP lens with 532 nm incident light. The 0 sector GZP lens behaves analogous to a FZP lens with multiple focal points, while the 8 sector GZP lens has only one focal point. Figure 2c illustrates the focal plane light distribution of the 8 sector GZP lens, and Fig. 2d shows the 3D version of the light distribution. A high spot contrast was observed, and the focal spot intensity was 1124 times higher than the average background.

**Figure 3:**
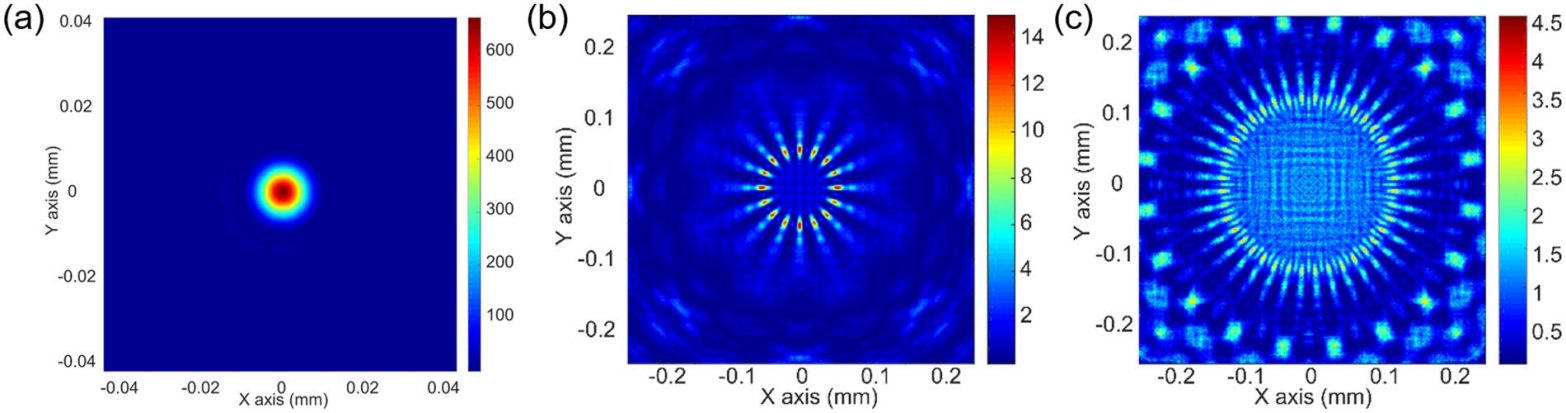
Simulations of the GZP lenses. (**a**–**c**) Intensity distributions at z = 1/3 *f* for 0, 8 and 20 sector GZP lenses when illuminated with an incident light of 650 nm.

The off focal plane (at axial distance of z = 1/3 *f*) light distributions of 0, 8 and 20 sector GZP lenses were also studied by modelling (Fig. 3a–c). For the 0 sector GZP lens, a higher order focal point was formed, while for 8 sector and 20 sector GZP lenses only doughnut-like images were observed. The azimuthal intensity distribution in the “doughnut” pattern (number of light peaks and valleys) was determined by the number of sectors. Thus, for GZP lens with sector numbers larger than 0, the higher order diffractions orders were present; however, they did not intersect at the optical axis.

**Figure 4:**
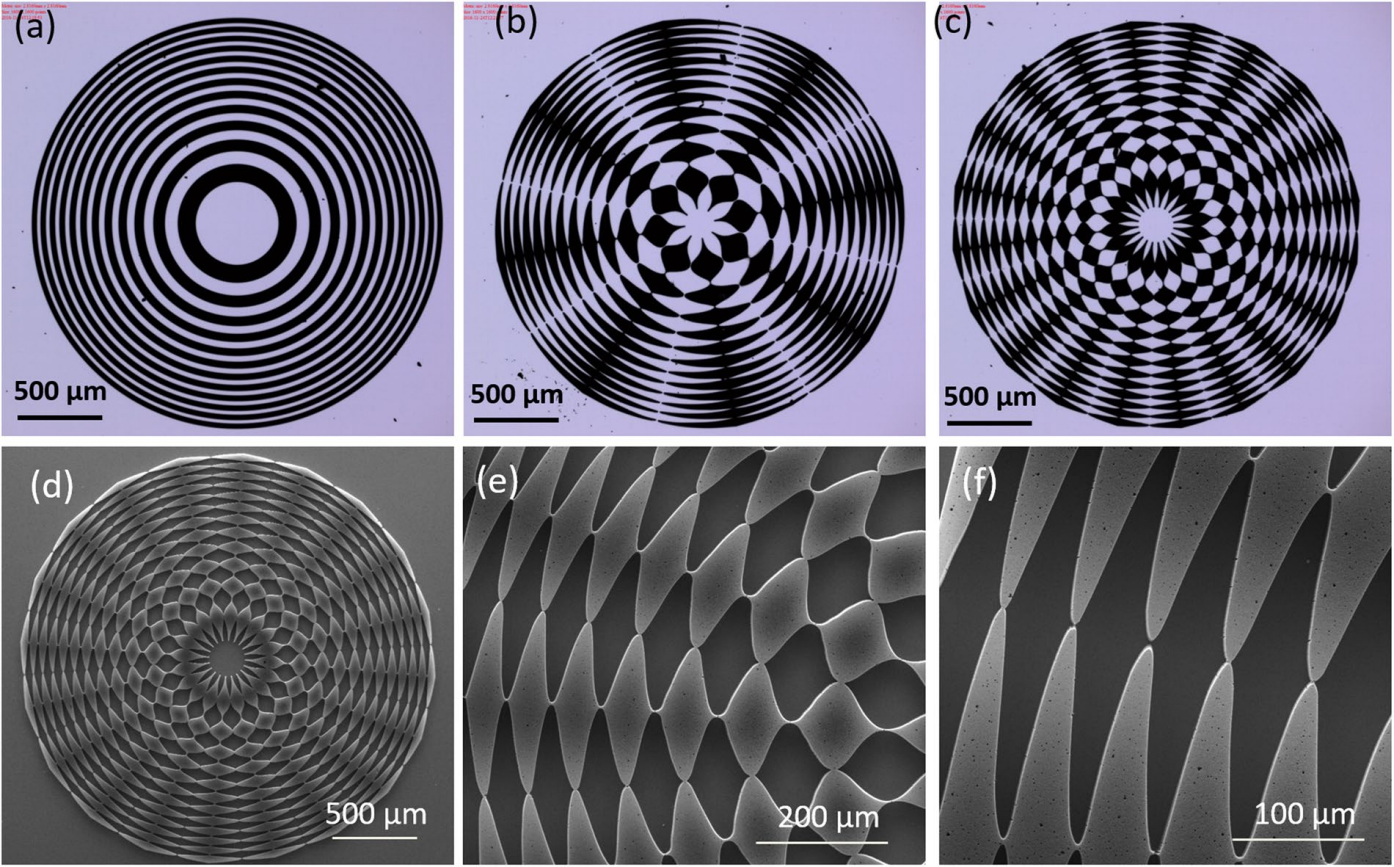
Microscopy images of the lenses. (**a**–**c**) Optical images of 0, 8 and 20 sector GZP lenses. (**d**–**f**) SEM images of GZP lens (20 sectors), with (**d**) A single GZP lens on silicon substrate, (**e)** and (**f**) are enlarged area of the GZP lens.

Figure 4 shows the optical images of three samples and Scanning Electron Microscopy (SEM) images of the 20 sector GZP lenses having defined edges and high resolution geometry.

### Optical Characterization of the GZP Lenses

**Figure 5:**
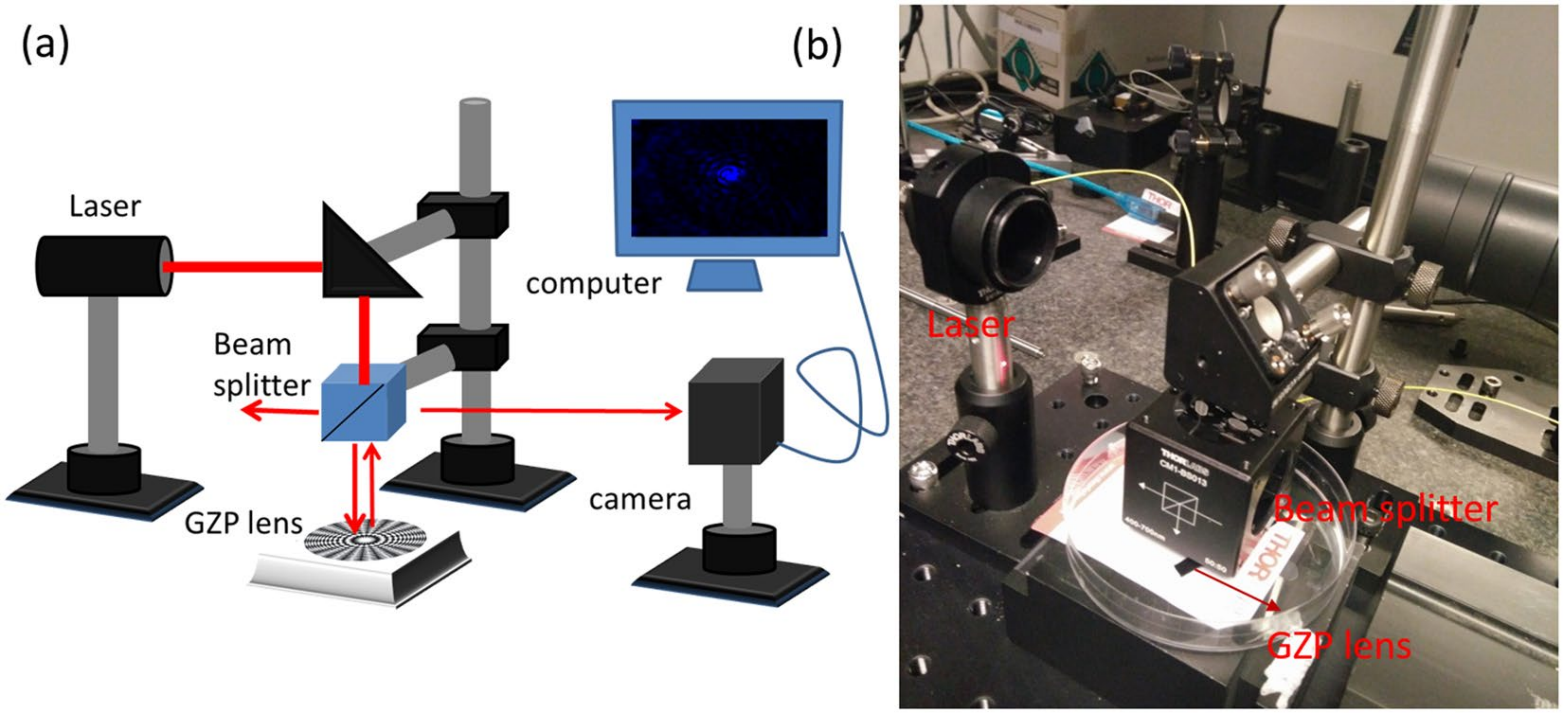
Optical characterization of GZP lenses. (**a**) Schematic and (**b**) photo of the experimental setup for optical measurement.

GZP lenses (0, 8 and 20 sector) were characterized by an optical microscope (Alicona Infinite) at a magnification of × 5 (Figs S1–3). The height difference between the neighbouring zones of the GZP lenses were ~25 µm. To observe the focusing effect of the GZP, an optical arrangement was setup for lens characterization in reflection mode (Fig. 5). A light beam from a diode laser (650 nm, 4.5 mW) was collimated and directed at the zone plate using a steering mirror and via a beam splitter (50:50). Light reflected from the GZP was partially reflected toward a digital camera (Carl Zeiss). The camera position was adjusted to obtain the focal point.

**Figure 6:**
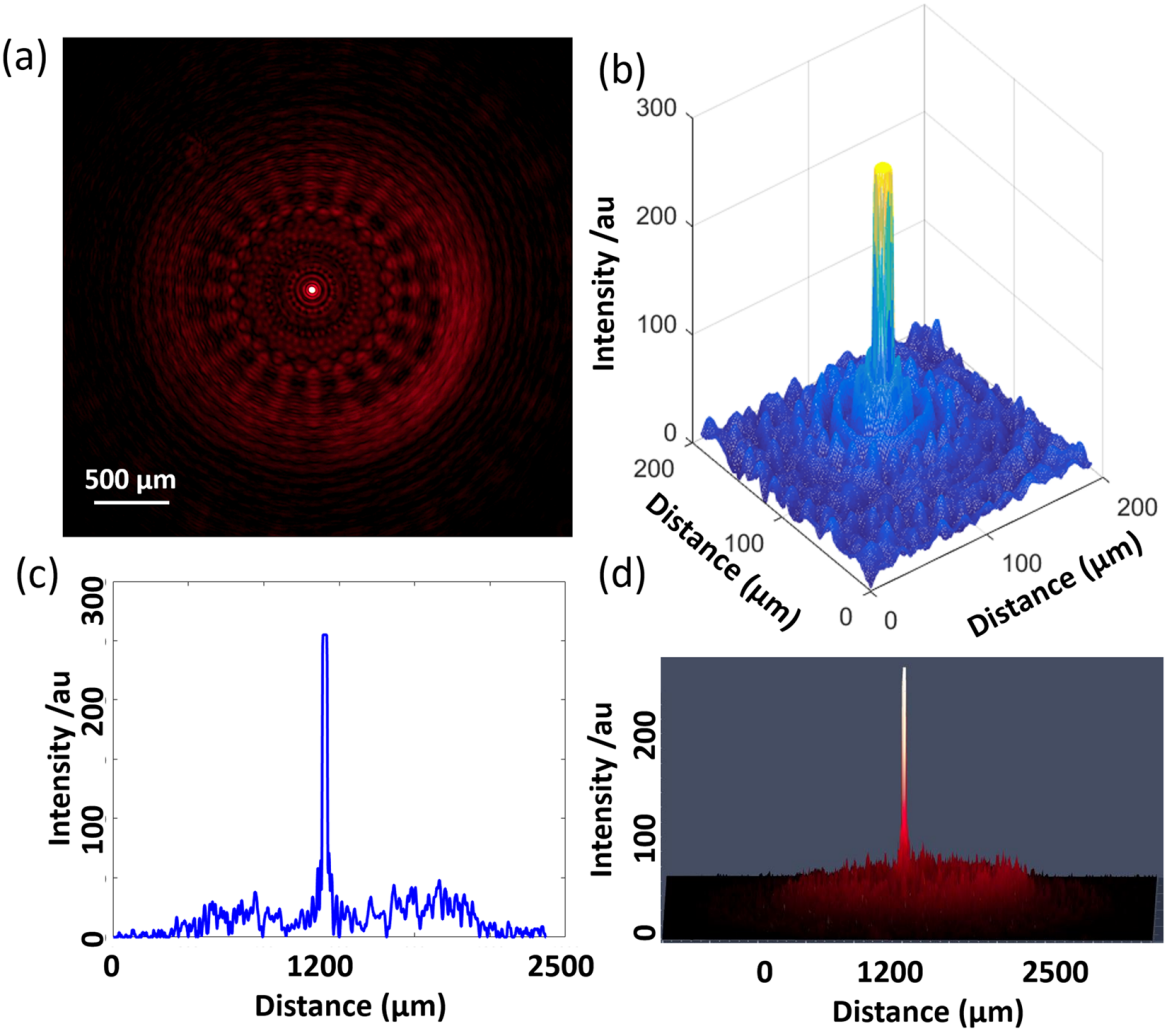
Optical detection of 20 sector GZP lens (**a**) Optical images of a 20 sector GZP lenses focusing 650 nm incident light with high contrast, (**b**) 3D image of the focusing area and (**c**) Horizontal cross light distribution via focal point, and (**d**) 2.5D images of (**a**).

Figure 6a shows a photograph of the high contrast focal point results of the 20 sector GZP lens with 650 nm red incident light. Figure 6b is the 3D focusing area of the GZP lens, while (d) is 2.5 D image of (a), which is obtained by the digital camera. Figure 6c illustrates the light distribution along the horizontal line via the focal point. All of the lenses had high lensing performance, as high contrast focal points could be seen clearly. In the present work, the 20 sector GZP lens were demonstrated because of its fabrication complexity. As images for GZP lenses of other sectors, can be seen in S4. The detected focal lengths were 9.9 cm and 11.8 cm for red and green light respectively, corresponding to the theoretical values of 9.84 cm and 11.75 cm.

The focal efficiencies and focal contrast of 0, 8, and 20 sector GZP lenses were measured as 5.62%, 4.06%, and 5.08%, respectively (incident light 650 nm) (Table 1). Since the absorption of CNT as well as the reflectance of silicon are not 100% as assumed in the simulation, the focal efficiencies were lower than the theoretical values. As show in Figure S5, the absorption of CNT array is 86.3% at 650 nm incident light. The absorption can be enhanced by controlling the CNT growth parameters to achieve a relatively low density array^16^. However, significant absorption appears in the UV regime, which reveals the advantages of CNT usage for shorter wavelengths.

**Table 1:** Focal efficiency, focal contrast, and signal noise ratio of 0, 8 and 20 sector GZP lenses with red (650 nm) incident light.

	Focal Efficiency			Focal contrast			Signal noise ratio		
**Sector**	0	8	20	0	8	20	0	8	20
**Red beam**	5.62%	4.06%	5.08%	459.5	381.2	487.7	69.31%	71.82%	66.73%

The focal point intensities of the 0, 8 and 20 sector GZP lenses were 459.5, 381.2 and 487 times of the average background. As for the signal to noise ratio, FWHM of the intensity of the curve was calculated via the focal point (e.g., curve in Fig. 6c) as compared to the total intensity of the entire curve. 0, 8 and 20 sector GZP lenses had high signal to noise ratio of 69.31%, 71.82%, and 66.73%, respectively.

**Figure 7:**
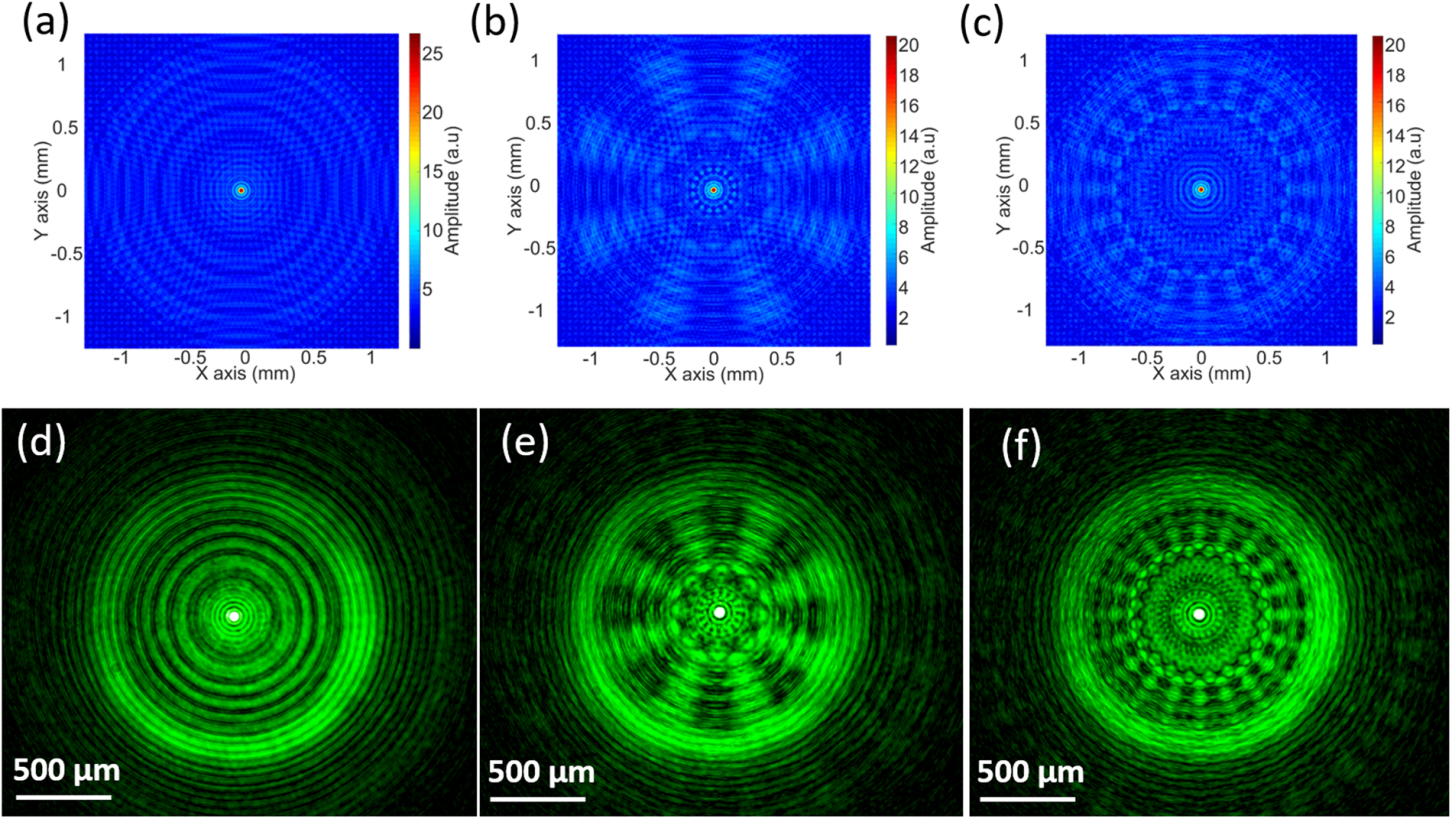
Focal plane light distributions of 0, 8, and 20 sector GZP Lenses. (**a**–**c**) Simulation results, and (**d**–**f**) experimental results with 532 nm incident light illumination.

While there were subtle differences between the pattern in Fig. 6a and the simulation result in Fig. 2c, there were similarities observed with the off focal plane diffraction in Fig. 3. The uniform blue background in Fig. 2c was due to the high focal contrast. Therefore, the square root of the intensity was (Fig. 7a,b,c) in order to convert the intensity into an amplitude to be comparable to the captured images from the experimental data (Fig. 7d,e,f). The corresponding experimental results were obtained under 532 nm incident light illumination (Fig. 7d,e,f), which were overexposed to show the low-intensity background pattern. Thus, the experimental results had agreement with the simulated models, allowing the predictability of the optical response. In addition, these patterns from off focal plane diffraction had low intensity as compared to the focal plane.

## Conclusion

We have developed novel CNT GZP lenses on silicon substrates using the high-absorption nanoscale surface roughness carbon nanotube forest. The lensing performance was simulated with Scalar theory of diffraction. Then, the lenses were fabricated by photo-lithography and CVD. The high lensing performance of the lenses were experimentally characterized to be 487.8 times focal point contrast. These CNT GZP lenses are a promising milestone in realizing highly-efficient optical components on silicon based electronics in creating miniaturized photonic chips.

## Method

### Principle of GZP lens

The light transmittance from a binary FZP lens with N zones is^17^:

1 $$t(r)=\{\begin{array}{c}\,1,\,\sqrt{m-1}{r}_{1} < r\le \sqrt{m}{r}_{1}\,(m=1,\,3,\,5\ldots )\\ 0,\,\sqrt{m}{r}_{1} < r\le \sqrt{m+1}{r}_{1}\,(m=2,\,4,\,6\ldots )\end{array}$$

where m = 1, 2, 3 …. N, and ${r}_{1}$ is the radius of the innermost zone. The Fourier expansion of t(r) for the FZP can be expressed as^5^:

2 $$t(r)=\frac{1}{2}+\frac{1}{i\pi }\sum _{p=-\infty }^{\infty }\frac{1}{p}\exp (\frac{-i\pi p{r}^{2}}{{r}_{1}^{2}}),\,p=1,3,5\ldots \ldots $$

Each term in the series, except the first one, has the same form as a thin lens. Thus t(r) has principle maxima at ${f}_{p}=\pm {r}_{1}^{2}/p\lambda$, $p=1,3,5\ldots$. In contrasts to the FZP lens, a GZP lens has a radially sinusoidal transmittance function^5^:

3 $$t(r)=\frac{1}{2}[1\pm \,\cos (\frac{\pi {r}^{2}}{{r}_{1}^{2}})]$$

In Eq. 3, the GZP lens has only a single pair of conjugate foci thus suppresses the higher-order diffracted light^17^. However, its applications have been limited by the fabrication difficulties in realizing such sinusoidal transmittance distribution^6^. The underlying idea is to rearrange the transmittance function t(r) in the azimuthal direction, *i.e*., t = t(r, θ). Thus, t(r) can be expressed as:

4 $$t(r)=\frac{1}{2\pi }{\int }_{0}^{2\pi }t(r,\theta )d\theta $$

Then the GZP lens will be composed by a series of transmitting arcs, and each arc having an angle ${\phi}_{W}$:

5 $${\phi}_{N}({r}_{j})=\frac{2\pi t({r}_{j})}{W}$$

where *r*_*j*_ ranges from 0 to the radius of the plate, and W is the number of arcs for radius ${r}_{j}$. The average integral of amplitude transmission over the circular zone at the radius r is equal to the desired transmission t(r).

### Computational Modelling of the GZP Lenses

Scalar theory of diffraction was used to determine the far-field solution of diffractive element. A MATLAB program was coded to map the intensity distribution along the axis of propagation as well as to estimate the theoretical diffraction efficiency of the lens to understand the optical characteristics of the proposed GZP lenses. Scalar theory of diffraction was used to determine the far-field solution of diffractive element rather than solving Maxwell’s equations as the exact solution was difficult to be obtained for the latter^18^. The far-field diffraction distribution could be calculated at any distance along the axis of propagation by solving Fresnel approximation of Rayleigh-Sommerfeld integral equation^19^.

6 $$g({f}_{x},{f}_{y})={\int }_{-\infty }^{\infty }{\int }_{-\infty }^{\infty }u({x}_{0},{y}_{0})\ast {e}^{(-i2\pi [({f}_{x}{x}_{0})+({f}_{y}{y}_{0})])}\,dx\,dy$$

7 $${f}_{x}=x/\lambda z\,{\rm{and}}\,{f}_{y}=\,y/\lambda z$$

where g (f_x_, f_y_) is the far-field energy distribution, and *u*(x_0_, y_0_) is the initial light field of energy. The maximum intensity at the focal plan for FZP and GZP can be calculated by Eqs 6–7, respectively^20^:

8 $${I}_{FZP}={(2N)}^{2}$$

9 $${I}_{GZP}={(\pi N/2)}^{2}$$

In the simulated models, it was found that if the number of zones of the GZP lens were the same, the intensity profile on the focal plane would be the same regardless of the incident light and number of sectors. The diffraction intensity of GZP was 61% of the intensity of FZP with the same number of zones Eqs (6–7). The theoretical efficiencies of the fabricated lenses were 10.13% (1/π^2^) for FZP, and 6.25% for GZP^20^. The intensity profile along the axis of propagation for FZP and GZP were^20^:

10 $${I}_{FZP}(p)={(\frac{\sin (N\pi /p)}{\cos (\pi /2p)})}^{2}$$

11 $${I}_{GZP}(p)={\sin }^{2}(\frac{\pi N}{p})[1+\frac{{p}^{2}}{{(1-{p}^{2})}^{2}}]$$

where p is z/focal length.

### Fabrication of the CNT GZP Lenses

Vertically aligned CNT forests were grown on a highly-doped n-type silicon substrate by chemical vapor deposition (CVD). The silicon substrate was first coated with a photo-lithography patterned Al (10 nm) / Fe (1 nm) multilayer catalyst, deposited by electron-beam evaporation. The substrate was then heated to 900 °C, at 10^−2^ mbar. During heating, gaseous ammonia was introduced to etch the surface of the nickel catalyst and stimulate the formation of nanoislands which template the induced nanotube self-assembly process. Acetylene was chosen as the carbon feedstock, and was introduced to the deposition chamber when the temperature reached 900 °C. The growth process lasted for 1 to 10 minutes resulting in 10 μm to 100 μm tall CNT forest. Following the growth process, the samples were annealed in hydrogen at 1000 °C for 2 h to remove amorphous carbon deposits and residual impurities.

The CNT forest had a thickness of ~100 μm (Figure S6). The magnified SEM picture in Figure S6b illustrates that the density of the CNT array are high, and the diameters of CNTs are 5–10 nm.

## Electronic supplementary material

Supplementary Material
