# Supplementary material for: Carbon Nanotube Array Based Binary Gabor Zone Plate Lenses
Source: Sci Rep. 2017 Nov 10;7:15256. doi: 10.1038/s41598-017-15472-9 (PMC5681564; doi:10.1038/s41598-017-15472-9)
Supplement: Supplementary file 1 — Supplementary Material [file 41598_2017_15472_MOESM1_ESM.pdf]

SUPPLEMENTARY MATERIAL

Carbon Nanotube Array Based Binary Gabor Zone Plate Lenses

Sunan Deng[1,2,*], Tahseen Jwad[1], Chi Li[3], David Benton[4], Ali K. Yetisen,[5] Kyle Jiang[1], Qing Dai[3] and Haider Butt[1,*]

[1]School of Mechanical Engineering, University of Birmingham, Birmingham B15 2TT, UK

[2] Laboratory of Applied Photonics Devices, School of Engineering, École Polytechnique Fédérale de Lausanne, CH-1015 Lausanne, Switzerland

[3] National Center for Nanoscience and Technology, Beijing 100190, China

[4] Aston Institute of Photonic Technologies, Aston University, Birmingham, B4 7ET

[5] Harvard-MIT Division of Health Sciences and Technology, Harvard University and Massachusetts Institute of Technology, Cambridge, MA 02139, USA

*e-mail: h.butt@bham.ac.uk; sunan.deng@epfl.ch

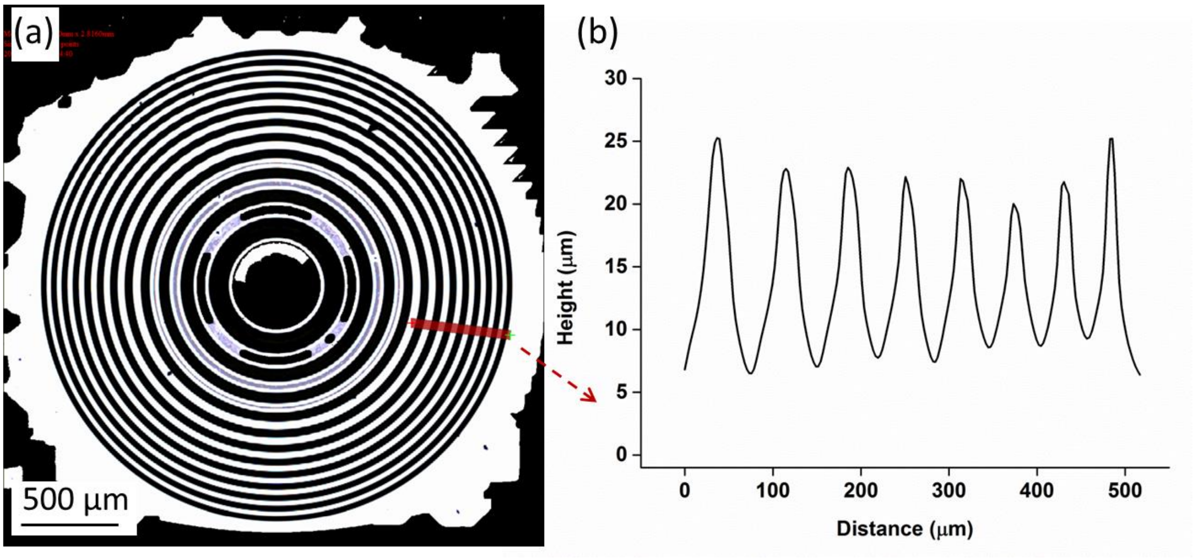

Figure S1. Optical characterization of the lens. (a) Optical image of 0 sector GZP lens, (b) height distribution along the red line in (a).

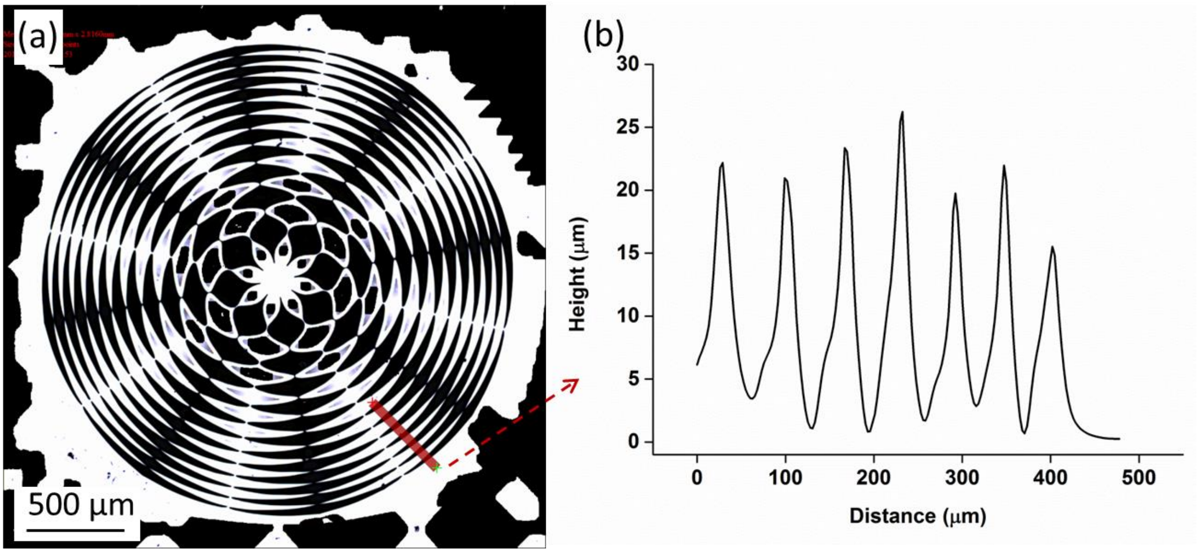

Figure S2. Optical characterization of the lens. (a) Optical image of 8 sector GZP lens, (b) height distribution along the red line in (a)

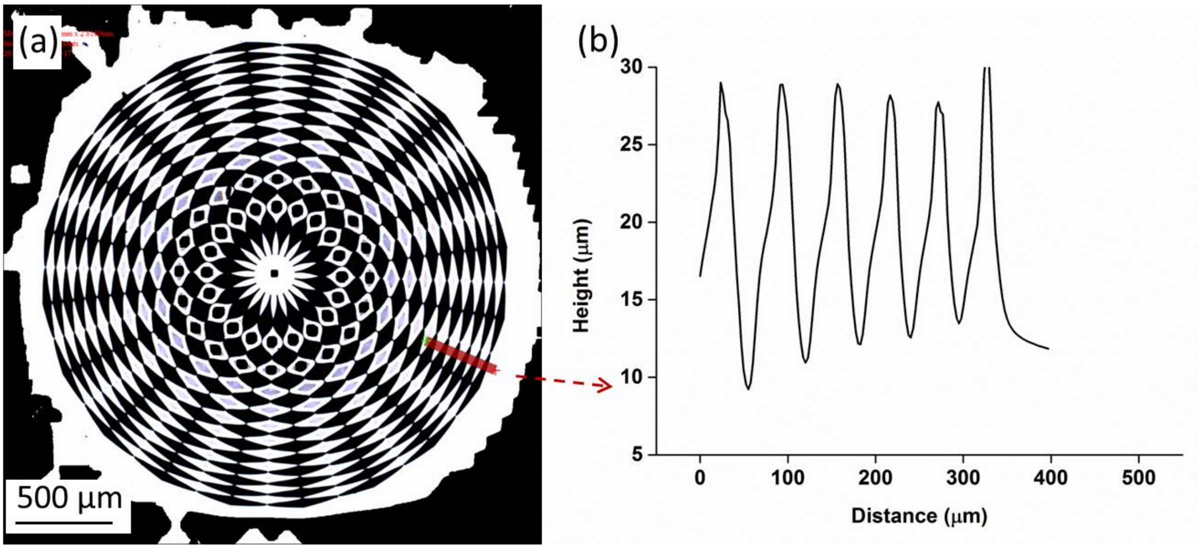

Figure S3. Optical characterization of the lens. (a) An optical image of 20 sector GZP lens, (b) height distribution along the red line in (a)

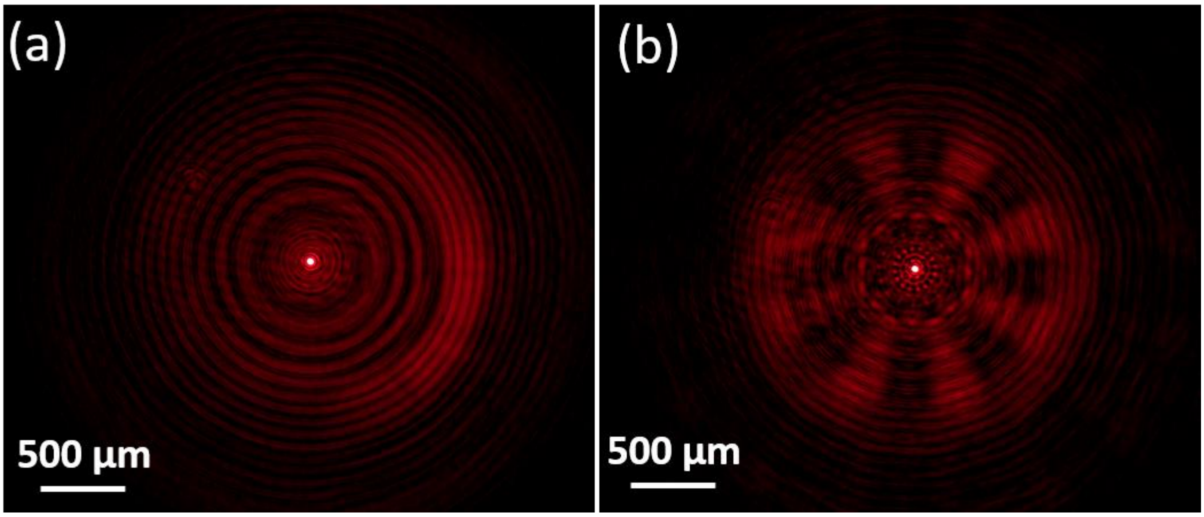

Figure S4. Optical images of (a) 0 sector and (b) 8 sector GZP lenses focusing 650 nm red light, taken by a digital camera (Carl Zeiss).

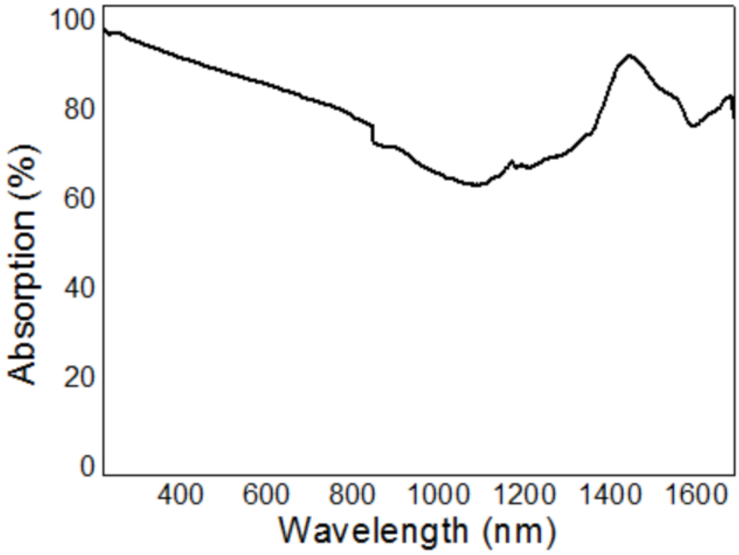

Figure S5. Absorption of vertically aligned carbon nanotube (CNT) forest sample.

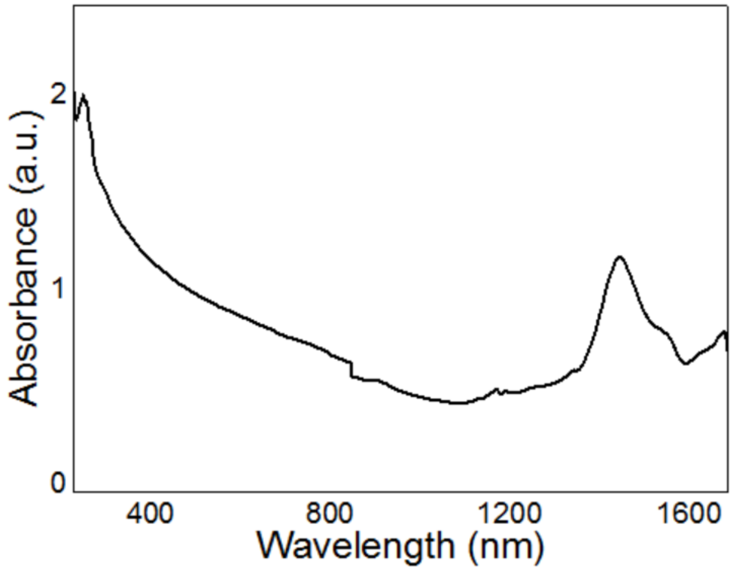

Figure S5. Absorbance of vertically aligned carbon nanotube (CNT) forest sample. The absorbance was measured by this equation: Absorbance=log(I_0/I_1), in which I_0 is the intensity of incident light while I_1 is the intensity of the light passing through the CNT sample.

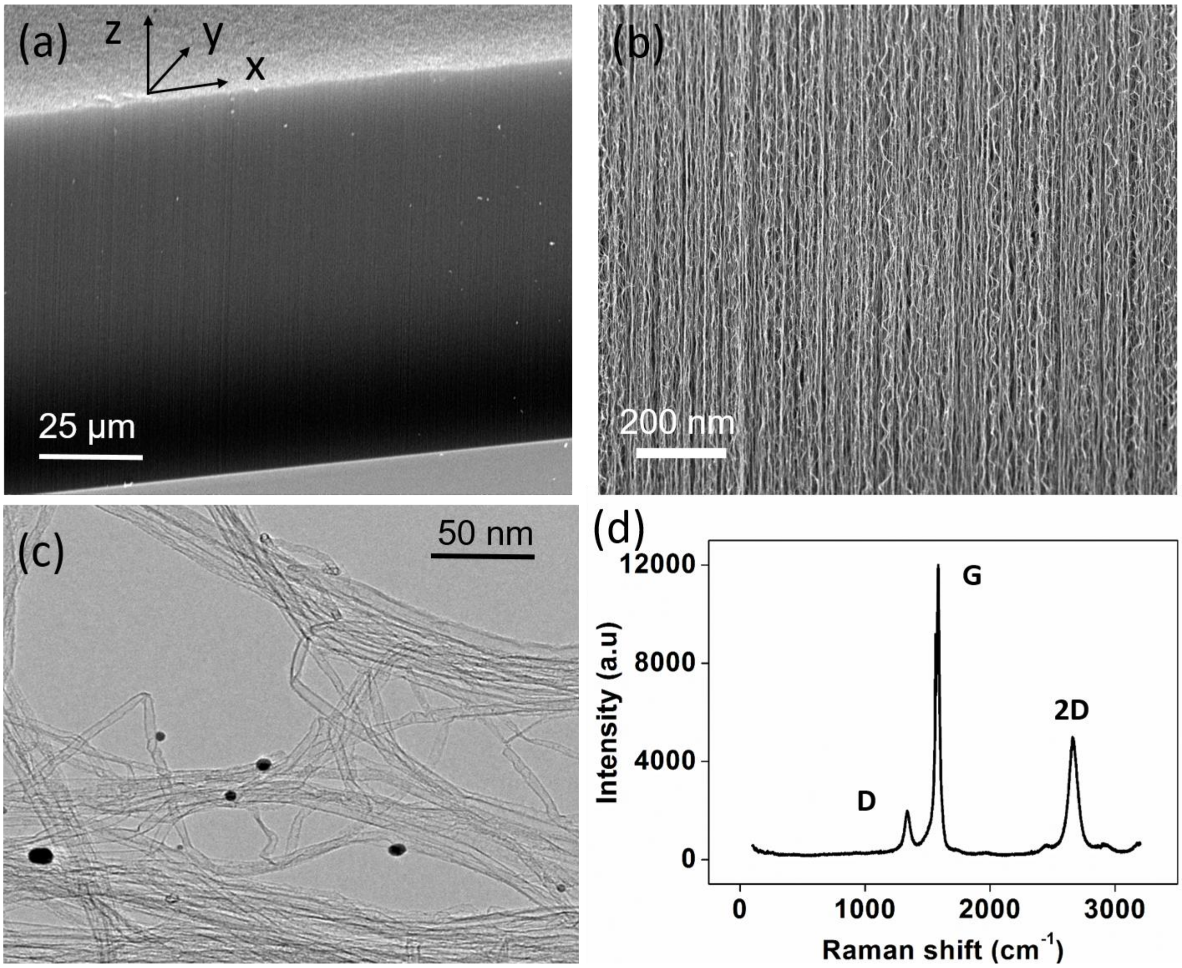

Figure S6. (a) Scanning electron micrograph (SEM) of vertically aligned carbon nanotube (CNT) forest sample. The height of the CNTs array is about 100 μm. (b) A side-view SEM image of the same sample at a higher magnification. (c) A transmission electron micrograph (TEM) of the sample, indicating that most of the nanotubes are with a diameter 5~10 nm. (d) Raman spectrum of the CNTs array. The D peak at about 1330 cm^{-1} stems from a structural defect of the graphite-like material, while the G peak near 1600 cm^{-1} is because of plane vibration in the sp^2 carbon materials. Therefore, the ratio of the intensities of G to D peaks (I_G/I_D) is usually used to evaluate the quality in graphitic carbon materials. By calculation, it is noticed that I_G/I_D of CNTs array is 6.24. The 2D peak at about 2660 cm^{-1} is a second order peak of the D mode; and the intensity I_{2D}/I_G is often used to estimate the thickness of graphene layers. Here the intensity ratio I_{2D}/I_G is 0.415.
